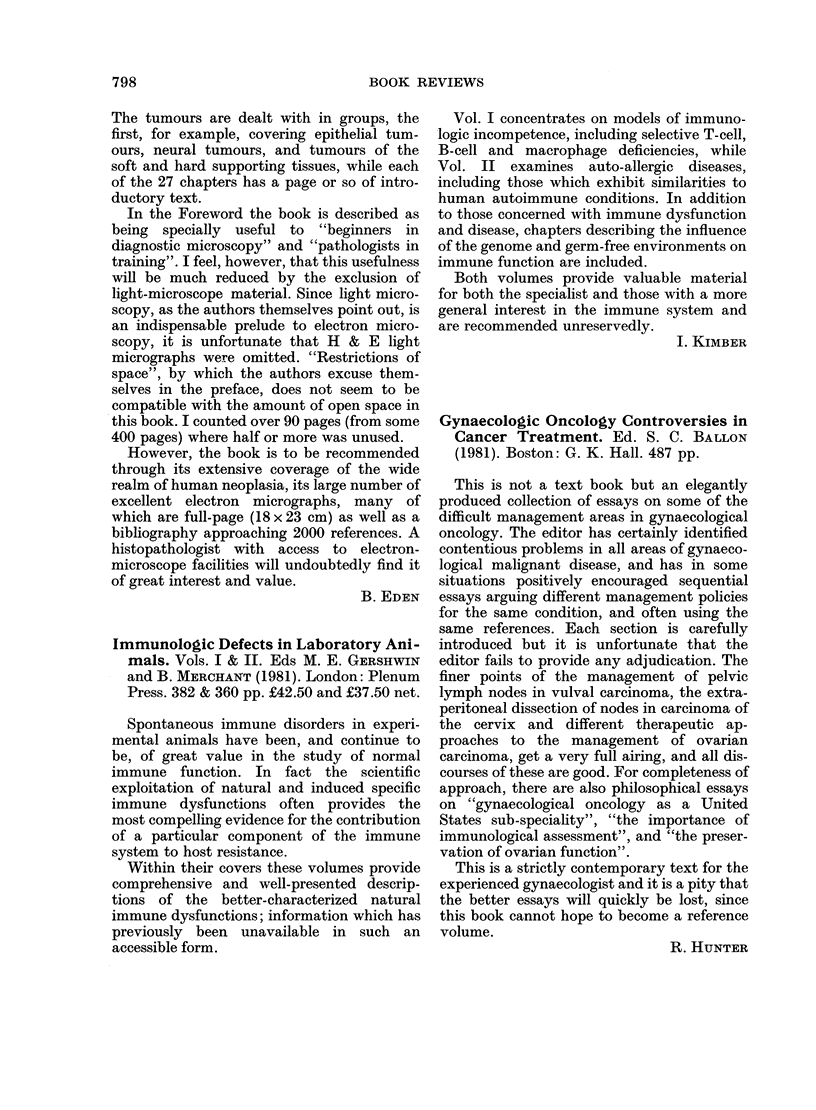# Gynaecologic Oncology Controversies in Cancer Treatment

**Published:** 1982-05

**Authors:** R. Hunter


**Gynaecologic Oncology Controversies in Cancer Treatment.** Ed. S. C. BALLON (1981). Boston: G. K. Hall. 487 pp.

This is not a text book but an elegantly produced collection of essays on some of the difficult management areas in gynaecological oncology. The editor has certainly identified contentious problems in all areas of gynaecological malignant disease, and has in some situations positively encouraged sequential essays arguing different management policies for the same condition, and often using the same references. Each section is carefully introduced but it is unfortunate that the editor fails to provide any adjudication. The finer points of the management of pelvic lymph nodes in vulval carcinoma, the extraperitoneal dissection of nodes in carcinoma of the cervix and different therapeutic approaches to the management of ovarian carcinoma, get a very full airing, and all discourses of these are good. For completeness of approach, there are also philosophical essays on "gynaecological oncology as a United States sub-speciality", "the importance of immunological assessment", and "the preservation of ovarian function".

This is a strictly contemporary text for the experienced gynaecologist and it is a pity that the better essays will quickly be lost, since this book cannot hope to become a reference volume.

R. HUNTER